# Mycogenic Nano-Complex for Plant Growth Promotion and Bio-Control of *Pythium aphanidermatum*

**DOI:** 10.3390/plants10091858

**Published:** 2021-09-08

**Authors:** Shaima M.N. Moustafa, Rania H. Taha

**Affiliations:** 1 Biology Department, College of Science, Jouf University, P.O. Box 2014, Sakaka 75471, Saudi Arabia; 2 Department of Botany and Microbiology, Faculty of Science, Minia University, El-Minia 61511, Egypt; 3 Chemistry Department, College of Science, Jouf University, P.O. Box 2014, Sakaka 75471, Saudi Arabia; 4 Department of Chemistry, Faculty of Science, Al-Azhar University, Cairo 11651, Egypt

**Keywords:** Zn-Nanocomplex, *Trichoderma longibrachiatum*, *Pythium aphanidermatum*, antifungal activity

## Abstract

(1) Background: biological way is one of the most ecofriendly and safe strategies for nanomaterials synthesis. So, biosynthesis-green method was used for the preparation of Zn(II) complex (in the Nano scale) from the reaction of the schiff base ligand 2,2′-((1E,1′E)-(1,2-phenylenebis (azanylylidene)), bis(methanylylidene))bis(4-bromophenol), and Zn(II)sulphate. The biogenic ZnNP-T was characterized by different methods. Our purpose was to evaluate the ability of biosynthesis-green method for the preparation of Zn(II) complex as an antifungal agent against diseases from fungal species. (2) Methods: in this work, isolates of *Pythium aphanidermatum* and *Trichderma longibrachiatum* were obtained, and *Trichderma longibrachiatum* was used to prepare nano metal complex. We tested the pathogenicity of nano metal complex against seedling and germination of seeds, and we evaluated the effectiveness of ZnNP-T for growth promotion of *Vicia feba* in early stage and inhibitory activity against *Pythium aphanidermatum*. (3) Results: antagonistic activity of ZnNP-T was tested in vitro against *Pythium aphanidermatum*, and then the growth rates of *Vicia faba* were determined. The obtained data revealed that mycelial growth of pathogenic fungus was inhibited about 73.8% at 20 ppm. In addition, improved the total biomass of *Vicia faba* in the presence of *P. aphanidermatum*. All concentration of ZnNP-T positively affected root weight of *Vicia faba* seedlings, and positively affected shoot weight. Root and shoot lengths were affected by using 20 ppm of ZnNP-T with up to 180 and 96.5 mm of shoot and root length compared to that of the control, while germination percentage was significantly enhanced with up to 100% increase after 72 h of germination. (4) Conclusion: one of the modern challenges in vegetable or fruit production is to enhance seed germination and to grow healthy plants with strong root system. In future, there should be a focus on using of biogenic Zinc nano-complex as plant growth promoter agents.

## 1. Introduction

There is an increasing demand to produce organic food, and the use of biofertilizers and biopesticides is the best way to achieve an alternative, ecofriendly production. In fact, *Trichoderma* spp. has the greatest resistant to pesticides and is categorized as an integrated control and good biocontrol agent [1,2] because of its ability to produce many enzymes, such as chitinases, glucanases, and proteases. In addition to biocontrol, *Trichoderma* spp. was recognized as plant growth promoter and a promoter of different plant defense mechanisms [1,3].

Nanoparticles have a very important role in the biotechnology industries [4] and is considered one of the fastest growing fields because of their biological, chemical and physical characterization. The biosynthesis of nanoparticles complex from fungi are a significant branch due to the fungi having the greatest tolerance and metal bioaccumulation capabilities. Biogenic methods for nanoparticles forming by plants [5,6], fungi [7,8], and bacteria [9,10] were previously described.

A great promise in this area is the Schiff bases. A Schiff base can be defined as the nitrogen analogue of aldehyde in which the carbonyl group is replaced by the azomethen group [11]. The transition metal complexes having O and N donor of Schiff bases ligands possess unusual configuration and structural liability, and they are also sensitive to the molecular environment [12]. The importance of the present study stems from the preparation of Nano Schiff base complex, its characterization, and its hopeful applications in different fields by using *Trichoderma longibrachiatum*, which is a nonpathogenic, fast-growing, and environmentally friendly fungus. In addition, we studied the properties of the biogenic nano metal complex and evaluated it against *Pythium aphanidermatum*, which causes dangerous diseases such as damping-off and root and stem rot disease in vegetables and fruits, and we also studied the effect of change in substituent on the biological properties of Schiff base derived from o-phenylenediamine and substituted salicyaldehydes.

## 2. Results and Discussion

**Figure 1:**
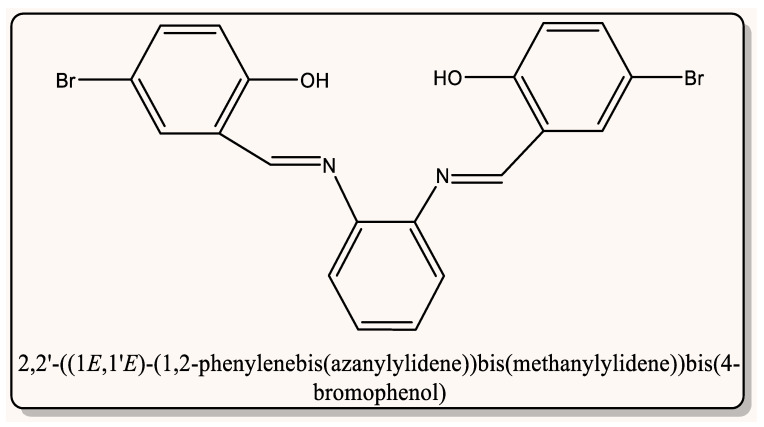
Schematic route for preparation of free ligand.

In the present study, the analytical data of the previously prepared Schiff base ligand and its Zn nano metal complex suggest the structures as in Figure 1. The Schiff base ligand and its Zn-nano complex were subjected to elemental analyses (C, H, N and O). The results of elemental analyses with molecular formula and the M.P. are in Table 1. The results obtained were well in agreement with those of the suggested formulae. The M.P. were sharp, indicating the purity of the prepared compounds. The results of the elemental analyses of the isolated complex agree with those required by the proposed formula of the complex.

**Figure 2:**
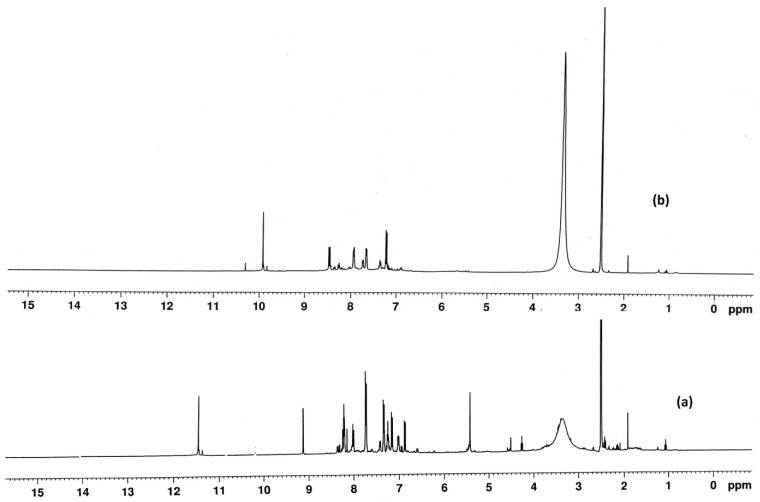
^1^H NMR data for (**a**) free ligand and (**b**) its Zn(II) nano-complex.

The NMR spectrum of the Schiff base ligand Figure 2 and Table 2 showed a singlet peaks at 9.335 ppm and at 11.247 ppm due to the azomethine and phenolic protons, respectively. In addition, multiplet signals at 6.465–8.175 ppm are attributed to the aromatic protons [13,14]. The comparison of the NMR data of the ligand and that of its Zn (II)metal complex confirms the mode of coordination between the ligand and its metal ion. By complexation, the spectrum of the nano-Zn complex displays a significant shift of the signals due to C=N group and OH protons, indicating the involvement of both OH as well as C=N groups in coordination to the metal ions without their deprotonation, suggesting that the ligand acts as neutral tetradentate ligand, O_2_N_2_ coordination sphere.

The IR spectrum of the ligand was compared with those of its metal complex to confirm the mode of bonding, as demonstrated in Table 3.

The infrared spectrum of the schiff base ligand has a broad absorption band at 3456 cm^−1^, which was due to the phenolic group [15,16]. The spectrum also displays medium band at 1290 cm^−1^ due to υ_C-O_ (phenolic group) [17]. Additionally, a strong band at 1620 cm^−1^ was observed due to υ_C=N_ of azomethine group [18]. By careful comparison of the spectrum of the nano-metal complex with those of the Schiff base ligand, it was found that the band at 3456 cm^−1^ shifted to lower frequency region at 3401 cm^−1^ in the metal nano complex, suggesting the involvement of OH group in complexation without its deprotonation. The strong bands at 1620 and 1290 cm^−1^ due to υ_C=N_ and υ_C-O_ (C=N and OH groups) are shifted to the lower wave numbers 1532 cm^−1^ and 1240 cm^−1^, respectively, in the metal complex, indicating the coordination of nitrogen and oxygen to the metal ion. Also, the spectrum of the complex shows three bands at 1401cm^−1^, 1372 cm^−1^, and 1025 cm^−1^ corresponding to unidentate coordination mode of NO_3_ group. All these results are consistent with the conductance data. Conclusive evidence of the bonding is also shown by observing new bands in the infrared spectrum of metal nano complex in low frequency region at 550 cm^−1^ and 515 cm^−1^, which may be attributed to υ_M-O_ and υ_M-N_, respectively, which are not observed in the spectrum of the Schiff base ligand [19,20].

### Mass Spectra

**Scheme 1:**
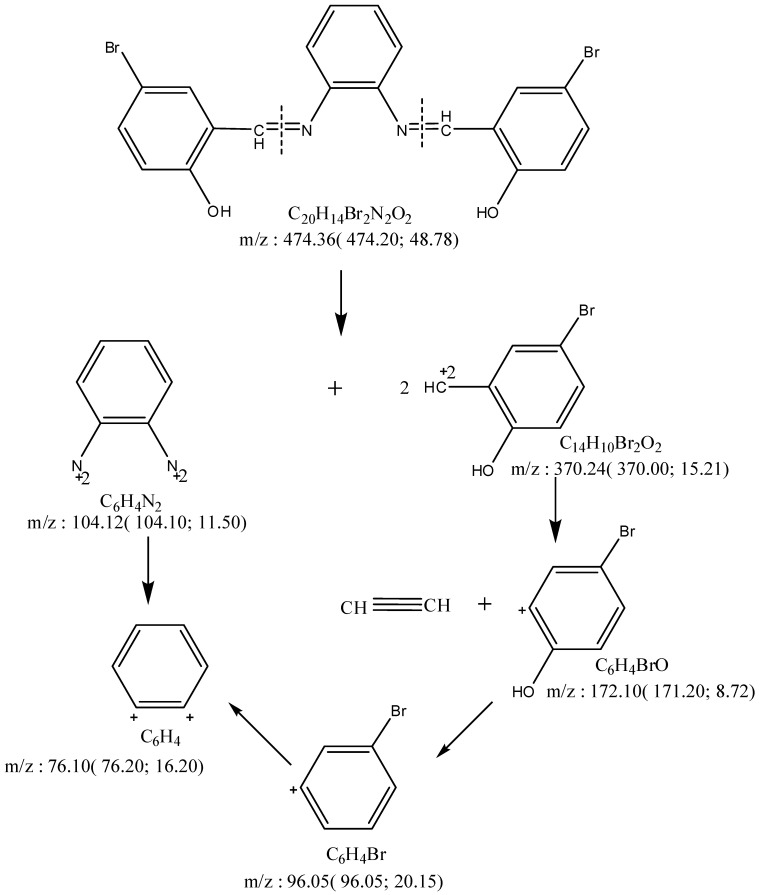
Mass fragmentation pattern for free ligand.

The mass spectrum of the free ligand shows the parent peak at m/e = 474.36 (48.78%) that agree with the molecular formula C_20_H_14_Br_2_N_2_O_2_. Also, the spectrum shows numerous peaks corresponding to various fragments, and their intensity indicates the stability of the fragments. Scheme 1 represents the proposed pathway for the decomposition steps for the ligand.

The conductivity Λ_m_ value of the Zn-nanocomplex can be calculated using the relation Λ_m_ = K/C, where C is the molar concentration of the nano metal complex solution and K is the specific conductance. The complex was dissolved in (10^−3^ M) DMF and the molar conductivity of the solution at 25 ± 2 °C are measured Table 1. The complex was found to have molar conductance value of 11.25 ohm^−1^mol^−1^cm^2^, indicating that this complex is nonelectrolytic in nature. Also, the values indicate the bonding of the nitrate ions to metal cations [19,21].

UV-vis spectra of the ligand and its metal complex was performed in DMF at room temperature at wavelength range 200–800 nm. The significant electronic absorption bands of the ligand, its nano complex, and the magnetic moments of the complex are given in Table 4. Two absorption peaks were observed in the spectrum of the ligand at 265 and 339 nm due to п-п* and n-п* transitions, respectively, and are attributed to benzene and the azomethine groups [20,22]. In the spectrum of metal complex, the absorption bands attributed to п-п* and n-п* transition were shifted to lower or higher frequency due to the coordination of the Schiff base ligand with the metal ions. Zn (II) complex is diamagnetic in nature with no *d*-*d* transition. So, according to all this obtained data, the octahedral structure may be suggested.

**Figure 3:**
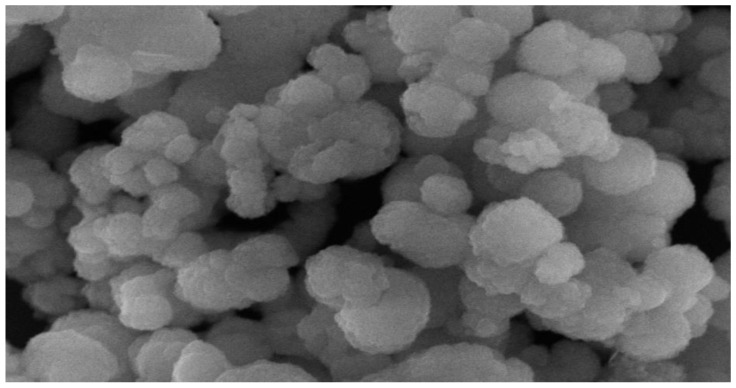
SEM image of nanoparticle complex.

SEM (Figure 3) studied the size and the morphology of the nano complex. The particles are semispherical in nature, with some agglomerations. The SEM images also revealed the stabilization of Zn(II) nanoparticles due to interaction with the Schiff base ligand [23].

**Figure 4:**
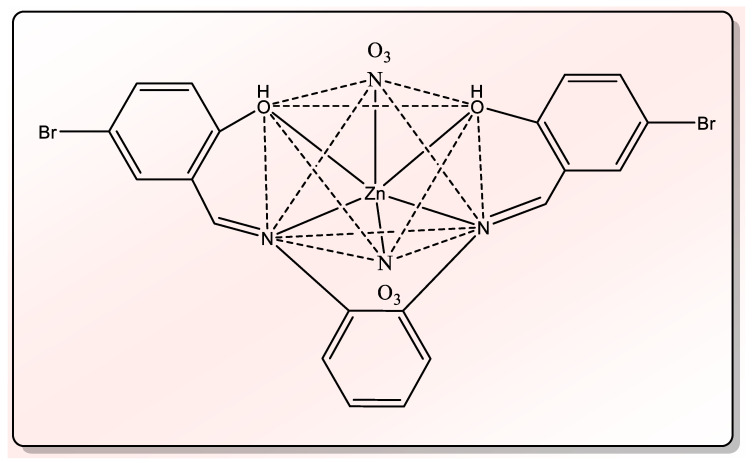
Suggested structure of Zn nano complex.

Correlating all these results, the structure of the complex can be suggested to be as demonstrated in Figure 4.

**Figure 5:**
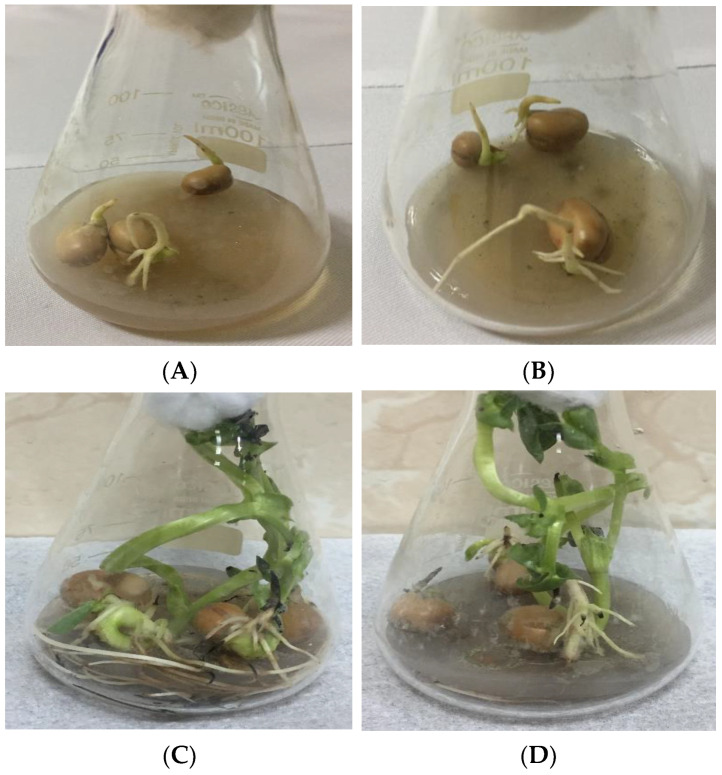
Effect of nano-metal complex on *Vicia faba* seedlings grown in Earlenmeyer flasks containing 2% water agar. (**A**) control after 4 days; (**B**) treated seeds with nano-metal complex (20 ppm) after 4 days; (**C**) control after 20 days; (**D**) treated seeds with nano-metal complex (20 ppm) after 20 days at 25 °C.

Fungi are the most efficient microorganisms when it comes to the biological production of Mycogenic Metal Nanoparticles synthesis [24]. This is due to fungi having enhanced processes for accumulating metals, and they also have capacity to produce a high number of bioactive metabolites, [25,26]. Fungi do not require complex nutrients; these are easy to grow and manipulate, and they also have high metabolites and production of biomass, as well as a high metal uptake and wall-binding capability [27,28,29]. Our Experiment revealed that nano-metal complex (20 ppm) was nonvirulent to *Vicia faba* germinating seeds, Figure 5.

**Figure 6:**
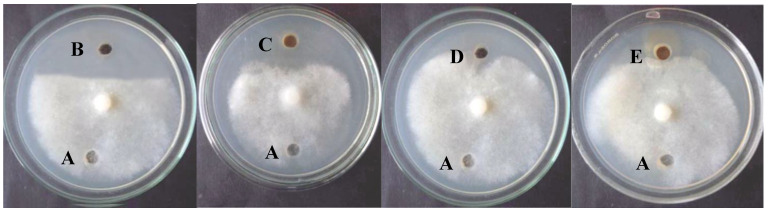
Inhibition of mycelial growth of *P. aphanidermatum*. (**A**) Control containing sterile distal H_2_O; (**B**) control containing Metalaxyl; (**C**) treated containing nano-metal complex (20 ppm); (**D**) treated containing nano-metal complex (15 ppm), and (**E**) treated containing nano-metal complex (10 ppm) on PDA at 26 °C for 6 days in the dark.

**Figure 7:**
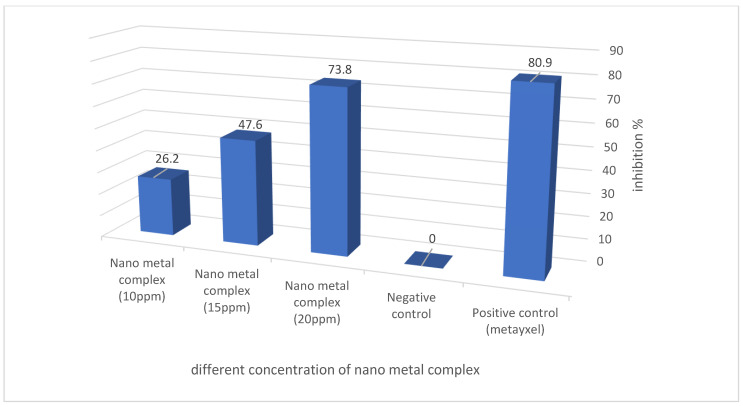
Inhibition % of *P. aphanidermatum* exposed to different concentration of nano-metal complex, on PDA at 26 °C for 6 days in the dark.

Measuring inhibition zones around each tested concentration of nano-metal complex, being determined against *P. aphanidermatum*, indicated that this different concentration manifested inhibition zones with different diameters around *Pythium* growth on PDA medium. Nano-metal complex (20 ppm) was the strongest antagonist (31 mm), followed by nano-metal complex (15 ppm) having a moderate antagonistic effect (20 mm), and nano-metal complex (10 ppm) was the lowest one (11 mm), and their inhibition percentages were about 73.8, 47.6, and 26.2%, respectively, when compared with that of the control Table 5 and Figure 6 and Figure 7. Biological control of damping-off diseases was successfully applied using *T. longibrachiatum* [30,31]. El-Sayed (2017) [32] reported that *Trichoderma* spp. is known to control all pathogens either directly by inhibition of sporulation and growth of the pathogen mechanisms, such as enzyme production and mycoparasitism, or indirectly by competing for nutrients and space, modifying the environmental conditions, or by promoting plant growth and enhancing plant defensive mechanisms and antibiosis.

Kamala and Indira (2011) [33] evaluated the activity of three *Trichoderma* isolates (T73, T80, and T105) regarding biocontrol activity of *P. aphanidermatum*. They also assayed the different biocontrol mechanisms, such as protease, chitinase, β-1,3-glucanase activity, cellulase, and production of volatile and nonvolatile compounds. Their results showed that *Trichoderma* (T-105) reduced the pre-emergence and postemergence damping-off disease incidence in infested soil with *P. aphanidermatum* and showed the highest disease control percentage under in vitro, as well as the pot experiment [34,35,36]. In our study, we evaluated the biogenic nano-metal complex in pot experiments, which reveal that data in Table 6 have differences in the germination %; the highest germination % of *V. faba* was observed after 72 h in nano-metal complex 20 ppm at about 100%, nano-metal complex 15 ppm at about 96.6%, and in 10 ppm germination, percentage was 90%. By comparison, the control was around 86.6%.

**Figure 8:**
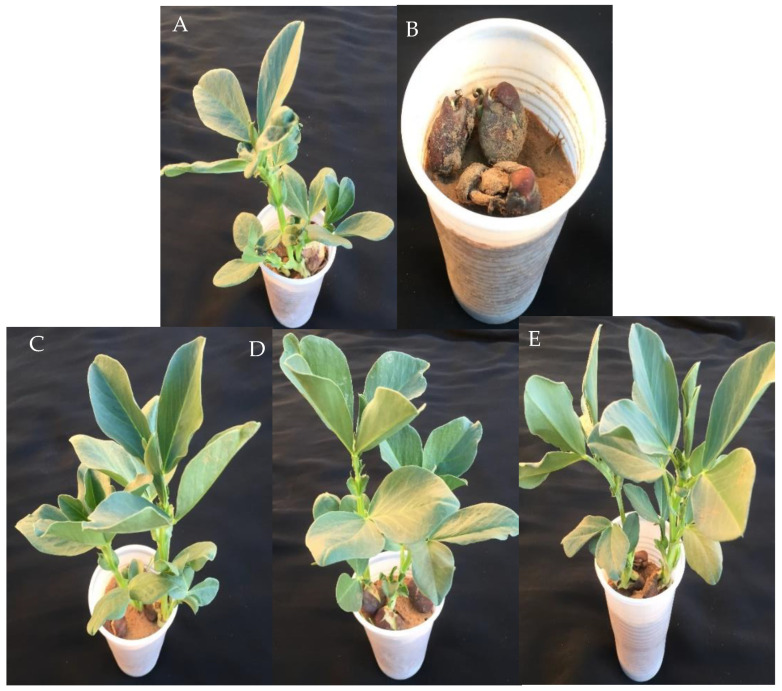
Effect of biocontrol agent and plant growth promotion of nano-metal complex on *Vicia faba* seeds germination in pots containing sandy loam soil cultivated with *P. aphanidermatum*. (**A**) Control seeds sown in free *Pythium* soil, (**B**) control seeds sown in soil infested with *P. aphanidermatum*, (**C**–**E**) and seeds sown in soil infested with *P. aphanidermatum* and irrigated with nano-metal complex (10, 15, 20 ppm), respectively, after 4 weeks.

**Figure 9:**
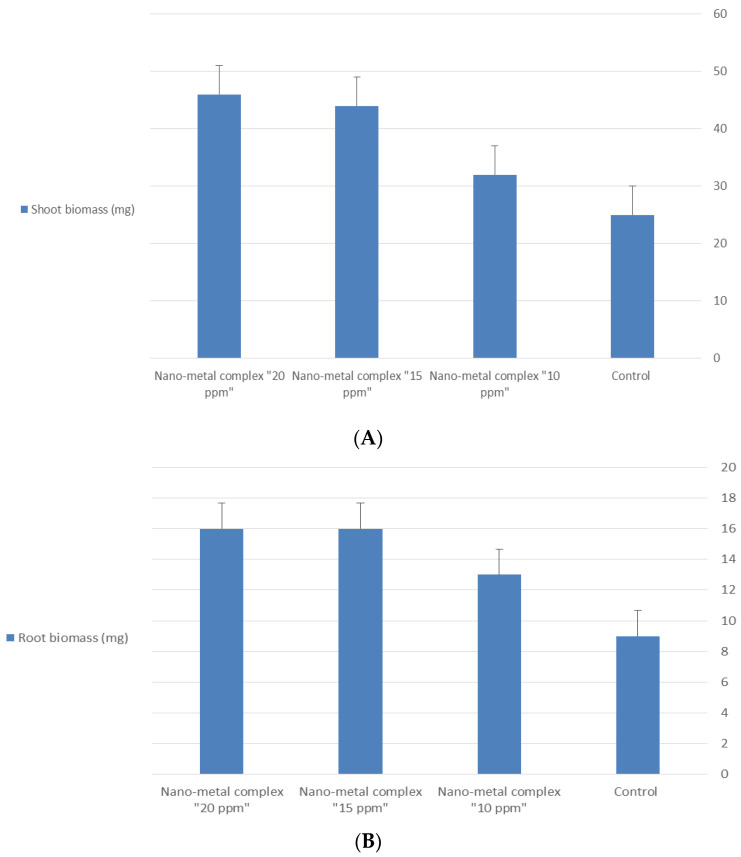

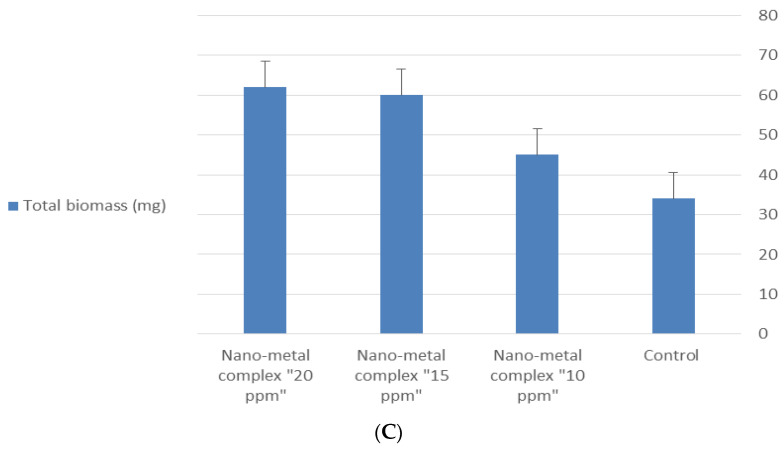
Effect of nano-metal complex on *Vicia faba* plant biomass after 4 weeks of planting. (**A**) Shoot biomass; (**B**) root biomass; (**C**) total biomass.

With observance to root and shoot lengths, results in Table 6 show that nano-metal complex had effects in both concentration 15 and 20 ppm for shoot length at about 180 mm, while the nano-metal complex (10 ppm) treatment recorded the lowest value for root length at about 150 mm. In addition, in the same concentration, the root lengths were about 95.2 mm and 96.5 mm, respectively. The nano-metal complex (10 ppm) treatment recorded the lowest value for root length about 81.8 mm, Figure 8 and Figure 9. From previous results, the nano-metal complex (20 ppm) had the best record in both germination as well as shoot and root length.

The increased applications of metal nanoparticles in several areas required new biocompatible, safe, and active nanostructures with less dangerous byproducts from synthesis reactions [37]. Mycogenic nanoparticles are observed as biocompatible, ecofriendly, less toxic, safe, and the cheapest alternative, with lowest consumption of energy and highest yields when compared with that of other physical or chemical synthesis [38,39]. While the advantages of metal nanoparticles as the best alternatives against antimicrobial species of pathogenic fungi and other microflora are well-known, many challenges and opportunities are ahead of us. Given that the effects of NPs result from a combination of multiple factors, the potential development of resistance against them is more difficult and less likely [40]. Future studies should be focus on testing new isolates, discovering new metal nanoparticles, and clarifying their structures and mode of action as antimicrobial agents. Novel discovery of mycogenic MNPs include the study of extremophilic and endophytic isolates. While the endophytic increased in relevance in recent years, the extremophilic is still a limited focus of study, as research is more concentrated on other purposes rather than their use against antimicrobial species [41].

## 3. Materials and Methods

### 3.1. Materials

In this study, all chemicals used were of highest purity. Organic solvents used included C_2_H_5_OH and (DMF), which were spectroscopic pure from British Drug House (BDH) London. Distilled water was collected from glass equipment. The other materials, such as 5-bromo-salicylaldehyde (Sigma), *o*-phenelendiamine (Aldrich, Saint Louis, MI, USA), and Zn(NO_3_)_2_ (Merck) were also used.

### 3.2. Instrumentation

Microanalytical determinations were carried out in the Cairo University, (Microanalytical Center) Cairo, Egypt. The IR spectra were recorded on a Perkinelmer spectrophotometer (400–4000 cm^−1^) (KBr technique). Proton-NMR spectra (DMSO-*d*6) were measured at a Pruker spectrophotometer, using TMS as an internal standard. The uv–vis spectra were recorded on a Perkin–Elmer spectrophotometer. Mass spectra were recorded with the aid of a Shimadzu using a direct insertion probe (DIP) at temperature range 50–800 °C. The nano-sized complex was characterized with (SEM) a scanning electron microscope.

### 3.3. Synthesis of the Schiff Base Ligand

A solution of 5-bromo-salicylaldehyde (4.02 g, 20 mM) in absolute ethanol (50 mL) was added to a hot stirred solution of *o*-phenylenediamine (1.08 g, 10 mM). This mixture was refluxed for 3 h in water bath and cooling, and then, the products formed were collected by filtration. Yield (87.4%, 90.2%); M. P; 240 and 200 °C, respectively (the ligand was synthesized in our previous work [42].

### 3.4. Studied Micro-Organisms

Isolates of *Pythium aphanidermatum* and *Trichderma harzianum* were obtained from biology Department, Jouf University, Sakaka, Saudi Arabia, KSA. Precultures of *Trichoderma longibrachiatum* (MT550032) and *P. Pythium aphanidermatum* were made by growing on potato dextrose agar (PDA) for 7 days at 26 °C in the dark.

### 3.5. Preparation of Biomass of Trichoderma Longibrachiatum (MT550032)

Two discs (5 mm in diameter) of *T. longibrachiatum* (MT550032) were inoculated into 100 mL PD broth in a 250 Erlenmeyer flask and incubated at 26 °C for 7 days in the dark. Mycelium mats were collected by using filter paper (Whatman No. 1) and washed three times with sterilized distilled water to eliminate any adhering media that may be present. Mycelium mats (3 g) were dried for biosynthesis of nanoparticles.

### 3.6. Pathogenicity Tests of Nano Metal Complex against Seedling and Germination of Seeds

To use nano-metal complex as a biocontrol agent, it must produce metabolic toxins that affect seed germination and plant growth. In sterilized petri-dish, sterile filter paper 9 cm was putted and wetted with 10 mL of nano-metal complex; hence, fifty seeds of *Vicia faba* were added in the dish and incubated at 20 °C until seeds germinated. Ten mL of sterile distilled H_2_O were used as control.

Pathogenicity was tested in water agar (WA) medium for seed germination [13]. One hundred mL of WA (3%) was decanted in Erlenmeyer flasks with 100 mL of nano-metal complex (20 ppm) and then sterilized. After *Vicia faba* seeds germinated and began to form radicles and plumules, we chose the vital seeds, and three seeds were planted in Erlenmeyer flasks. All flasks were incubated in growth chamber at 25 °C with 12 h photoperiod for 4 weeks.

### 3.7. Biosynthesis of the Nano-Metal Complex

The Zn nano complex was prepared by mixing the Schiff base ligand (1 mM) and 1 mM of Zn (II) nitrate in the presence of *T. longibrachiatum* powder as a reducing agent as a novel method for the preparation of Zn-nanocomplex. The mixture was ground with a pestle in an open mortar at room temperature. The melted mixture was then allowed to solidify. The solid was filtered off and crystallized twice-using C_2_H_5_OH to turn Zn-nanocomplex into a pale brown crystal.

### 3.8. Bioassay of Nano-Metal Complex against Pythium aphanidermatum

The antifungal activity of nano-metal complex, biosynthesized by *T. longibrachiatum*, was examined against *P. aphanidermatum* by using the agar well-diffusion method. In petri dishes containing 15 mL of PDA media, one disc of *P. aphanidermatum* (3 mm) was inoculated in the center of each petri dish. In both edges of petri dishes, the wells of 3 mm diameter were made by using cork borer. One hundred µL of different concentration of nano-metal complex (10, 15, and 20 ppm) were poured in one well, and in anther well, 100 µL of sterile distill water were poured as a negative control, while Metalaxyl (100 µL) was used as positive control. All petri dishes were incubated at 26 °C in the dark. After 6 days, all the treatments were carried out in triplicates. Inhibition zones (mm) were measured, and percentage inhibition of fungal growth was calculated using the Equation (1):

Inhibition (%) = ((D − M) ÷ D) × 100(1)

where D is the growth diameter of *P. aphanidermatum* in the control (ppm) and M is the growth diameter of *P. aphanidermatum* in presence of nano-metal complex (ppm) [38].

### 3.9. Evaluation of Nano-Metal Complex as Antifungal Activity and Plant Growth Promotion in Pot Experiment

The ability of nano-metal complex in different concentrations (10, 15, and 20 ppm) along with its antifungal and growth enhancement effects on *Vicia faba* seedlings in soil infested with *P. aphanidermatum* were tested. For preparation of *P. aphanidermatum* infested soil, the propagule suspension of *P. aphanidermatum* was added at 30 propagules/g soil to the autoclaved soil. Thirty seeds of *V. faba* in the same size were used for each treatment. The surface of the seeds was sterilized with 2% sodium hypochlorite for 3 min and washed by sterile distilled H_2_O for 5 min, and then dried using sterile filter paper. Pre-germination test was done to select viable seeds.

The experiment was classified into five groups. In the first group, there was free *Pythium* soil was irrigated with sterile distilled water, while in the second group, soil infested with *Pythium* and irrigated sterile distilled water. In the remaining three groups, the soil infested with *Pythium* in each group and soil were irrigated with nano-metal complex-solution with different concentrations (10, 15, and 20 ppm). Pots were kept in a growth-illuminated cabinet at 25 °C with 12 h photoperiod under humid conditions. Soil moisture content was preserved at 35%. Experiments were performed with ten replicate pots per treatment. Germination % was calculated after 48 and 72 h by following Equation (2):

(2) $$Germination\;percentage=\frac{Number\;of\;germinated\;seeds}{Total\;number\;of\;seeds}\;\times 100$$

After 7 days of germination, seedling growth was determined in terms of shoot and root length (cm).

## 4. Conclusions

Zink nano complex was obtained using novel green method in the presence of *T. longibrachiatum* powder as a reducing agent. The composition of this complex was described by several physicochemical methods, suggesting an octahedral geometry around the Zn (II) ion. *Pythium aphanidermatum* causes dangerous diseases such as damping-off and root and stem rot disease in vegetables and fruits. The susceptibility of pathogenic fungi to Zn-nanocomplex-T synthesized by *T. longibrachiatum* was investigated. The results showed that the Zn-nanocomplex-T had antifungal activity against *Pythium aphanidermatum.* Therefore, our study opens perspectives for further exploration of fungal biogenic nanoparticles for use with the control of agricultural biocontrol.

## Figures and Tables

**Table 1:** Elemental analysis and some physical measurements of free ligand and its metal nano complex.

Compd. No. Empirical Formula	M.P. (°C)	Color (Yield %)	(%) Found (Calcd.) *Λ*m *			
			C	H	N	
**Free Ligand(L)** **C_20_H_14_Br_2_N_2_O_2_**	200	Dark Yellow (90.20)	36.89 (50.64)	2.93 (2.98)	12.01 (5.91)	**-**
**(5)[(Zn)(L)](NO_3_)_2_** **C_20_H_14_Br_2_N_4_O_8_Zn Nano Complex**	>350	Dark Yellow (85.79)	33.59 (33.90)	1.95 (2.00)	7.86 (7.91)	11.25

* ohm^−1^cm^2^mol^−1^, M.P. refer to melting point.

**Table 2:** ^1^H NMR data for Schiff base ligand and its nano-complex.

Compd. No.	δ_ph_	δ_CH=N_	δ_OH_
**L**	6.465–8.175	9.335	11.247
**Zn Nano Complex**	6.451–7.684	8.252	10.210

δ_ph_ for the phenyl, δ_CH=N_ for the azomethine and δ_OH_ for hydroxyl group.

**Table 3:** Infrared spectral data of free ligand and its metal complex.

Compd.No.	ʋ_O-H_ (Phenolic)	ʋ_CH=N_ (Azomethine)	ʋ_C-O_ (Phenolic)	ʋ_M-O_	ʋ_M-N_	Additional Bands
**L**	3456	1620	1290	-	-	-
**Zn-nanocomplex**	3401	1532	1240	550	515	1401, 1372, 1025 (coord.NO_3_)

ʋ_O-H_ vibrational of phenolic group, ʋ_CH=N_ vibrational of azomethine group.

**Table 4:** Magnetic moment and electronic spectral data of ligand and its Zn-nano complex.

Compd. No.	μ_eff_ (B.M.)	Absorption Bands (nm)		
		п-п*	n-п*	*d*-*d* Transition
**L**	-	265	339	-
**Zn-nano complex**	Diamagnetic	276	323	-

μ_eff_ (B.M.) indicating the magnetic susceptibility in Bohr magneton.

**Table 5:** Antifungal activity of nano-metal complex against *P. aphanidermatum* on PDA medium at 26 °C for 6 days in the dark.

*Pythium* spp.	Diameter of Inhibition Zone (mm)				
*P. aphanidermatum*	Positive Control (Metalaxyl)	Negative Control (Sterile Distilled H_2_O)	Nano Metal Complex (20 ppm)	Nano Metal Complex (15 ppm)	Nano Metal Complex (10 ppm)
	3.4 * ± 0.2 **	0	31 ± 0.4	20 ± 0.13	11 ± 0.41

* Diameter of inhibition zone (mm) ** Standard error of three replicates.

**Table 6:** Effect of nano-metal complex treatments on germination (%) and root and shoot lengths (mm) of *Vicia faba*.

Treatment	Germination % after 48 h	Germination % after 72 h	Length (mm)	
			Shoot	Root
Control	73.33	86.6	130	69.5
Nano-metal complex “10 ppm”	73.33	90	150	81.8
Nano-metal complex “15 ppm”	86.67	96.6	180	95.2
Nano-metal complex “20 ppm”	86.67	100	180	96.5

## Data Availability

The data presented in this study are available upon request from the corresponding author.
